# Making molecules work – stories of supramolecular translation

**DOI:** 10.1039/d5sc90071a

**Published:** 2025-07-29

**Authors:** Anthony P. Davis, Arri Priimagi, Matti Virkki, Jennifer R. Hiscock, Calden N. Carroll, Michael M. Haley, Darren W. Johnson, Jonathan F. Arambula, Krystle Karoscik, Jonathan L. Sessler

**Affiliations:** a School of Chemistry, University of Bristol Cantock’s Close Bristol BS8 1TS UK Anthony.Davis@bristol.ac.uk; b Smart Photonic Materials, Faculty of Engineering and Natural Sciences, Tampere University P.O. Box 541 FI-33101 Tampere Finland arri.priimagi@tuni.fi; c School of Natural Sciences, University of Kent CT2 7NH UK J.R.Hiscock@kent.ac.uk; d Department of Chemistry & Biochemistry and Materials Science Institute, University of Oregon Eugene OR 97403-1253 USA dwj@uoregon.edu cstimpso@uoregon.edu haley@uoregon.edu; e INNOVOTEX, Inc. 3800 North Lamar Blvd. Ste. 200 Austin TX 78756 USA jarambula@INNOVOTEXinc.com; f Department of Chemistry, The University of Texas at Austin 105 E. 24th Street-A5300 Austin TX 78712-1224 USA

## Abstract

Commercialising supramolecular chemistry has proved challenging, but encouraging stories are beginning to emerge. In this editorial we present accounts of programs aimed at diabetes management, humidity sensing, antimicrobials, shock absorbing materials, nitrate sensing and anti-cancer agents. The experiences of the authors are intended to help others following similar paths, assisting efforts to develop real-world applications of functional molecules.

## Abstract 2

Commercialising supramolecular chemistry has proved challenging, but encouraging stories are beginning to emerge.

## Graphical abstract

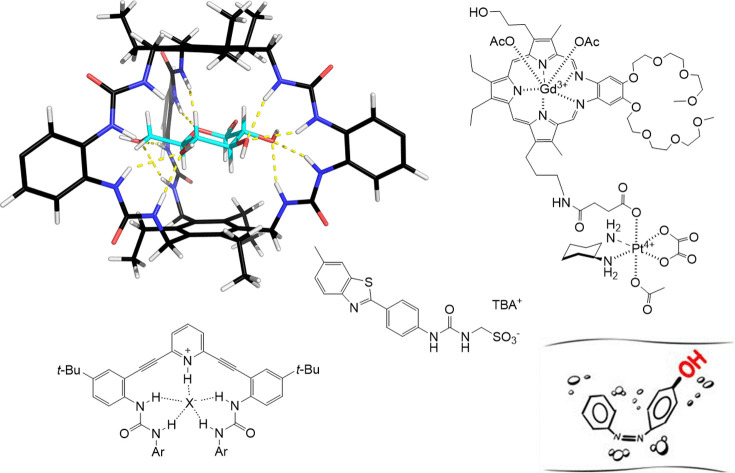

## Introduction

(Anthony P. Davis)

Supramolecular chemistry is not so easy to define, but perhaps the most satisfying (and inclusive) option is “the design and synthesis of functional molecules”. Supramolecular chemists (or at least most of us) want to make molecules that do something. One might imagine that discoveries in an area so defined would often progress to applications, but in practice this is not so common. A system needs to fulfil a need which is not satisfied in any other way; it must work in a complex real-world environment; and it must be economically viable. There is also perhaps a danger that the “proof-of-principle” is the interesting part which yields the glamorous paper. Optimisation for real-world applications may be less rewarding for an academic career, yet still too risky for industry.

Nonetheless, it is widely recognised that supramolecular chemists should try to apply their work to the world’s problems, especially if we want the world to keep funding our efforts. As the subject matures, we are better positioned to achieve success, and there are now a good number of positive examples. This perspective contains five stories outlining attempts to convert supramolecular chemistry into products for the real world. They vary in developmental maturity and in the level of conventional (financial) success, but all teach useful lessons. All end on an optimistic note, even if hopes are not quite fulfilled at this stage. We hope they will encourage others to take up the challenge.

## Ziylo and Carbometrics

(Anthony P. Davis)

### Diabetes and glucose recognition

Diabetes is a major medical problem. World-wide, there are estimated to be ∼540 million people living with the condition, of which ∼10% are Type 1 (producing no insulin themselves) and the remaining ∼90% are Type 2 (having lost sensitivity to insulin).^1^ Both types can be managed, but issues remain. In particular, all Type 1 diabetics need to inject insulin to keep their glucose levels down, and insulin is also required by many Type 2 diabetics. However, the process is not so easy to manage. The right amount of insulin must be injected at the right time, usually based on blood glucose analysis by a “finger prick” test kit. Too much insulin results in excessively low glucose levels (hypoglycaemia), which can be fatal. Patients may err on the side of caution, injecting too little so that their glucose levels are too high. In the long term this causes a range of problems; increased risk of cardiovascular disease, kidney disease, eye disease, and neuropathy sometimes leading to amputations.^2^ Any development which makes insulin treatment safer and easier can cause widespread benefit (and, considering the market size, could be very profitable).

Supramolecular chemists may see an opportunity here. Glucose is obviously central to diabetes, and the ability to sense and respond to glucose concentrations could be the key to improved treatments. Molecules that bind glucose selectively under biological conditions can serve as the basis for glucose sensors, which can help diabetics manage their insulin dosage, or glucose-responsive molecular switches which can be used to make insulins with activities that vary according to need. However, the design of glucose receptors is not straightforward. A fundamental problem is that carbohydrates in general are not only hydrophilic, but also “hydromimetic” – festooned with OH groups, they bear a close resemblance to clusters of water molecules. An effective receptor must be able to reject water in favour of the carbohydrate, and this is not easy.

**Fig. 1:**
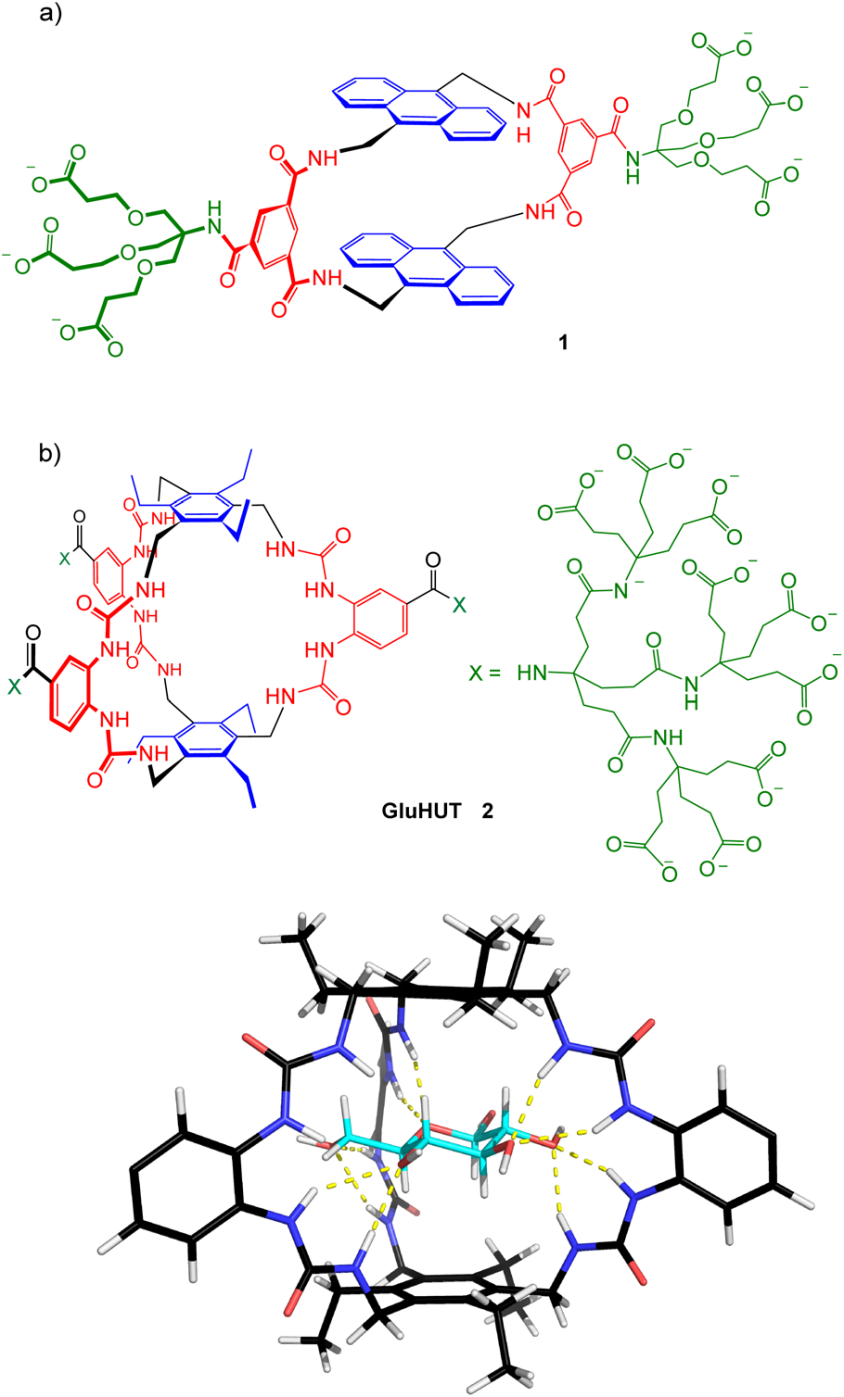
(a) The monocyclic glucose receptor 1 which led to the foundation of Ziylo. (b) GluHUT 2, the glucose receptor that really works, with a model of its complex with β-glucose.

We have been interested in binding carbohydrates in general, and glucose in particular, for many years.^3^ For most of this time, it seemed quite unrealistic to hope that we would ever produce anything useful. For the first 15 years or so, we were happy enough to bind glycosides in organic solvents – aqueous media seemed far too difficult as a starting point. When we did finally enter water, we were able to report glucose binding with *K*_a_ = 9 m^−1^ surely one of the lowest affinities to headline a paper in a decent journal.^4^ It wasn’t until some years later, in 2012, that we obtained the first hint of a practically useful result. The bis-anthracenyl macrocycle 1 (Fig. 1) was designed and made by Chenfeng Ke, then a post-doc in my group, and was found to bind glucose with *K*_a_ = 60 m^−1^ in water.^5^ Although this affinity is still modest, it is nicely calibrated to detect glucose in the range of medical importance (∼2–12 mM); any higher affinity and the receptor would saturate too early in that range. Moreover, the anthracene fluorescence increased quite substantially on binding, providing a built-in signalling mechanism. We weren’t sure how to turn this into a practical glucose monitor for people living with diabetes, but it made sense to patent before publishing. The University needed persuading – where’s your business case? – but eventually agreed. Clearly there was a chance it would come to nothing, but initial patents are inexpensive and when you’re aiming for diabetes the risk is worth taking.

### The birth of Ziylo

In fact, scepticism was merited – there were serious problems with macrocycle 1 as a practical glucose sensor. Nonetheless Harry Destecroix, a finishing PhD student in the group, was determined to start a company and saw our receptor as an opportunity. Harry is a natural entrepreneur with a positive outlook, and he was convinced that the problems could be solved. He put a few details into a UK government website, paid £12 and Ziylo was there. Other directors were myself and Harry’s friend Tom Smart, who knew something of accountancy. Very soon we were joined by my brother-in-law, Keith MacDonald, who brought extensive business experience and (importantly) money. Keith knew that the work was relevant to diabetes, and this was all he needed (see above).

Harry went to the University and started talking about the patent for 1. The situation was unusual because Ziylo already existed, with an established shareholder structure, so the standard spin-out format did not apply. On the other hand, the patent was not going anywhere else, so the University agreed to a deal. Ziylo started work, renting a fume hood in my laboratory, but Harry soon decided the rates charged by the university were too high and decided to build his own laboratory to host Ziylo. The result was “Unit DX”, an incubator laboratory for chemistry-based start-ups with 16 laboratories, 24 fume hoods, a 400 MHz NMR and much else. Ziylo moved in and was soon joined by other young companies, many of which were spun out of the University.

### GluHUT saves the day

While Unit DX was prospering, Ziylo was faltering – no way could be found of employing 1 in a practical sensor. The company had no income, and costs had to be covered. This is where fortune smiled. Back in the University another PhD student, Rob Tromans, was working on a new design, receptor 2, a fairly radical departure from our previous efforts. Modelling of 2 suggested that it would be highly effective and selective for glucose, and when Rob made it in October 2016 this proved to be the case.

“GluHUT”,^6^ as we later decided to call 2, bound glucose with affinities ∼100 times higher than previous efforts, and almost perfect selectivity.^7^ It seemed to have real commercial potential, and in Ziylo we had the ideal vehicle for the purpose. The University agreed, Ziylo paid for a patent and took over the development. Andy Chapman joined the company as CSO and oversaw the development of a scalable synthesis (with key input from Mike Orchard who, in an earlier life, had been one of my first PhD students). Thus armed, Harry, Keith and Andy went looking for customers.

**Fig. 2:**
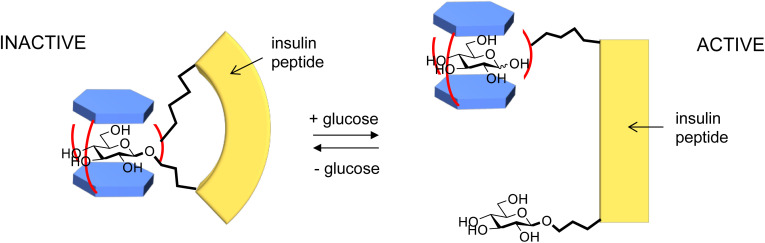
A concept for glucose-sensitive insulin (GSI). A glucose receptor and a glucosyl unit are attached at different points to the insulin peptide. In the absence of glucose they come together, rendering the insulin inactive due to a change in conformation (or some other reason). In the presence of glucose, the glucosyl unit is displaced and the insulin becomes active.

A key question at this stage was “what is it worth?” This is very difficult to answer if you are an academic, or even a businessman from outside the pharmaceutical industry. Ziylo had the good sense to engage consultants with specific knowledge of the diabetes business, and with their help we discovered that GluHUT was really quite valuable. A key application was in glucose-sensitive insulin (GSI, see Fig. 2). Several companies had been pursuing this concept (or something similar), but a suitable glucose receptor had not been found. GluHUT seemed well placed to fill the gap. In 2018 a deal was concluded with Novo Nordisk, the world’s major supplier of insulin, which greatly outstripped anything we might have imagined when GluHUT was first discovered. The details are confidential, but the limiting value was more than $800 million depending on regulatory and commercial milestones.^8^

As part of the deal, a new company Carbometrics^9^ was founded to work with Novo Nordisk on developing a GSI, and to apply GluHUT in glucose sensing. They have been able to develop glucose sensors with optical output, high selectivity and good long-term stability, and have a number of ongoing commercial collaborations. We can hope that GluHUT-based glucose sensors will be on the market before long. Meanwhile, Novo Nordisk and Carbometrics have reported a first example of a GluHUT-based GSI with evidence that it can reduce the risk of hypoglycaemia in animals.^10^ Unit DX has been rebranded Science Creates Incubators, has opened a second building, twice as large as the first, and has recently announced plans to build a third. Harry has expanded into venture capital with SCVC, who are currently raising for their 2nd fund with a target of $100m (he is also advising the UK government on support for spin-outs). A range of other services are provided under the Science Creates umbrella.^11^ Altogether, starting a deep tech business in Bristol is a lot easier than it was before Ziylo’s breakthrough.

The story of Ziylo is not exactly typical. Undoubtedly, we were lucky that GluHUT worked so well in an area where the target market is extremely large – the global market for insulin was valued at $18.7 billion in 2022. Nevertheless, there are lessons learnt which should be relevant to others. One is to protect intellectual property (IP) with care and especially to avoid premature disclosure. This can be difficult. You have an idea about an application, and some preliminary results which point in the right direction. Often you don’t really believe it will work in the real world, so you publish without worrying. But if you do eventually succeed, you may realise you have revealed too much. The invention has been disclosed in general terms, and a broad patent is no longer possible – only a few variants can be protected, and someone else can work round this. We were fortunate in that we suspected the potential of GluHUT from the outset, and were careful to reveal nothing. Secondly, it is vital to have the right mix of people. An academic, an energetic ex-student, and an experienced business head is probably ideal. Thirdly be ready to seek and pay for expert advice, especially when negotiating an exit.

Altogether this has been a rewarding experience. Over more than three decades we progressed from purely academic research through a vague hope of applications to genuine prospects for real-world impact. It would have been easy to give up at various stages, but we didn’t and persistence paid off. It wouldn’t have happened without the efforts of many collaborators and coworkers, and my thanks are due to all of these, especially those listed under Acknowledgements at the end of the article.

## OPTOSENSE – there and back again

(Arri Priimagi and Matti Virkki)

OPTOSENSE is a project name for the development of an azobenzene-containing thin film sensor for optical monitoring of the relative humidity of air. The measurement technology is based on real-time observation of the thermal *cis*–*trans* isomerization rate within the thin film which, using pre-defined calibration curves, can be converted to relative humidity at known temperature. It can provide a wireless, cheap, and relatively simple yet reliable alternative to capacitive-type humidity sensors, and operate in environments where electric readout is challenging or impossible. Hence, the project offers a straightforward path towards commercialization of basic research findings. Or does it?

After several years of applied research, OPTOSENSE has not reached commercial status. But it has, perhaps permanently, changed the ways of approaching the research findings in our group. Herein, we would like to share some highlights from our commercialization journey and what we learned along the way. The journey starts with a failed experiment which, thanks to a persistent PhD student working on it, turned to a research direction none of us could have anticipated. It continues with a talented postdoctoral researcher who took the operational lead of the subsequent sensor development and outreach, and the struggles he faced. And it ends with some tips from the group leader perspective on what to do and what not when aiming at research commercialization. Enjoy the ride!

### A little bit of history

Since the very beginning, my (Arri Priimagi) personal scientific journey has been (over)loaded with azobenzenes, the faithful photoswitchable companions which I tend to propose for a plethora of research ideas in materials applications. The story of OPTOSENSE begins in late 2013 or early 2014 with one such idea, executed by an outstanding PhD student Mikko Poutanen (grad. 2018) who navigated his way through the hurdles piled on his way by a concept that in hindsight – to put it mildly – was more complicated than it looked. The target was to (i) prepare microphase-separated block copolymers with domain size so large that they would act as Bragg reflectors at visible wavelengths, and (ii) supramolecularly (*via* hydrogen bonding) attach hydroxyazobenzene molecules to the poly(4-vinylpyridine) (P4VP) blocks. The eventual goal was to utilize azobenzene photoswitching to reversibly tune the reflection wavelength, or structural colour, of the periodically self-assembled structures. It was known from the earlier work of Prof. Olli Ikkala, my scientific mentor and Mikko’s co-supervisor, that (i) will work.^12,13^ But, as it turned out after 6–12 months of trial and error by Mikko, (ii) failed. This killed the scientific novelty of the whole project, causing sleepless nights for the PhD candidate and hair loss for me.

There were several reasons why the project failed, one of them being the surprisingly short *cis*-lifetime of the hydroxyazobenzenes when hydrogen-bonded to P4VP. Delving deeper into the photochemistry of the P4VP–hydroxyazobenzene complexes, we learned that their thermal *cis*–*trans* isomerization rate was highly dependent, *e.g.*, on azobenzene concentration and their packing into organized smectic layers.^14^ Frustratingly, the observations were extremely difficult to reproduce, varying literally day by day. The only solution to the reproducibility problems was to abandon the practical ease of working under ambient conditions and conduct the experiments in an environment with constant temperature and humidity.

In 2017, several years after Mikko started his PhD, it was clear that in thin hydroxyazobenzene-containing polymer films, humidity plays a pivotal role in the thermal isomerization kinetics. The last step of Mikko’s PhD was to study how systematic this dependence is. As it turned out, at all the temperatures investigated, there seemed to be an exponential (or at least close-to-exponential) relationship between the thermal isomerization rate and the relative humidity in the materials we focused on.^15^ The exponential relationship is important as it offers relatively straightforward means for converting the isomerization rate into relative humidity, if the temperature is known. This provoked us to ask whether the observations made could form the basis for an azobenzene-based humidity sensing device that would be useful in real-life applications.

It was time to get in touch with innovation services, file an innovation disclosure, and start considering issues related to IP protection.

### From fundamental to applied research – and back again

Towards the end of 2017 it became clear that OPTOSENSE forced us to step out of the comfort zone of blue skies research and set our minds towards applications. For funding the now clearly applied research, we turned to the European Research Council (ERC). I had an ongoing ERC Starting Grant, which made me eligible to apply for the ERC Proof of Concept (PoC) Grant. The objective of this funding instrument is to explore the commercial (or social) innovation potential of ERC-funded research ideas in a concise, 18-month funding period, and it seemed to perfectly fit our needs. But how to understand the market needs? We had a solution, but the problem that is addressed by our solution was far from clear. To find our *niche* within the humidity sensing market and to justify why our project should receive funding, we decided to collaborate with a consultant company who did the initial market research and helped us with the non-scientific parts of the proposal. This turned out to be the right solution as we got the funding on our first try. In Autumn 2018, the ERC PoC project OPTOSENSE was ready to launch.

At this point, Mikko had taken a position in a Finnish chemical company, and a new person, Matti Virkki, took over. Matti had followed the final parts of the work done by Mikko and was interested in getting involved by supporting the PoC project. Facing a possible premature end for the story, he decided to take the main responsibility. Matti had accrued a good mixture of laboratory experience in optical measurement setups and thin film sample fabrication during his PhD studies, but none in commercialization of research findings. Thanks to Matti’s efforts, proof-of-concept, in the form of an independent device capable of measuring relative humidity, was realized and shown to operate accurately while running continuously for six weeks. Together with MSc student Jani Patrakka, Matti also continued screening sensing layers for optimal response speed, sensitivity, sample-to-sample reproducibility, and long-term stability. The third part of the PoC project was a market survey performed by the consultants who had already helped during the preparation phase. The PoC project was finished in spring 2020 and the 18 months spent on it were successful and encouraging and we were eager to dig deeper. For this, additional funding was needed.

Business Finland (BF) is a Finnish Research & Development funder which, among other goals, prepares research-driven product or service ideas for commercialization *via* a funding instrument called “Research to Business” (R2B). R2B funding seeks to close the gap between research too applied to fit academic funding, but not yet proven enough to be adopted for product development at companies. With the help of the training and support offered by Tampere University Innovation Services, the application was successful on the second try, and the OPTOSENSE R2B project officially started in the beginning of 2021 – this time with a bigger team.

In an R2B project the work should be divided roughly equally between applied research and commercialization efforts. To reflect this, a team of four people was assembled. Matti took the operational lead and coordinated the project, with the aim of growing into the role of future deep-tech developer. Other members of the team were a business developer with first-hand experience in founding a deep-tech-based start-up company, a person to focus on market research and company outreach, and a materials developer to continue the sensing layer optimization. The second-generation portable sensing device was developed through outsourcing, since the know-how required for the integration of electronics, photonics, and embedded software, was too much to be handled in a lab equipped for fundamental research. At this stage we also made the biggest mistake along our commercialization path: the advanced concept device should have been created at an earlier stage, instead of collecting as much market information as possible to guide the device development prior to its fabrication. Seeing is believing, and even a non-optimized portable device would have been helpful in crystallizing our technology to potential customers. One-pagers and slideshows don’t do the job!

At the end of the R2B phase (early 2023) the OPTOSENSE story constituted an operational handheld device and sticker-type sensors with multiple materials differing in their optimal range for humidity/temperature. The device was piloted in monitoring the drying of concrete with purposely built sensor plugs, and the results were compared to a gold standard commercial device used for such monitoring. The device performance for this application was validated, yet it did not provide a clear enough benefit compared to existing, well-established technologies. We are still convinced that the most promising applications lie elsewhere but have failed to identify within the fragmented humidity sensing market the right starting point that would have enabled us to enter the game and only gradually create the all-round revolution we believe the technology could enable. After taking the concept from technology readiness level two to six, Matti moved on and started at the Technical Research Centre of Finland as a senior scientist in optical measurement device development – a position for which OPTOSENSE provided the perfect training.

Now, after several years of applied research, OPTOSENSE has been taken back to basics. Important learnings were that the humidity sensor market is large and growing, but also highly scattered. This means that there is an overwhelming number of possible opportunities and room for new innovative players. Furthermore, through our own journey it became clear how vital it is to refine the information from market reports and how to pitch an idea to funding parties, skills that would be useful to teach during undergraduate studies. Sami Vesamäki, whom we hired as the materials developer for the R2B project, continues to reveal the secrets of hydroxyazobenzene-containing polymer thin films as a PhD student.^16^ Thanks to the lessons learned, he will be able to approach his PhD topic from a point of view that would not have been possible in the group before OPTOSENSE. There is still much more to learn. Yet one important lesson we have learned is that we don’t have to know everything to aim for technology transfer – seeing the potential, believing in yourself, and understanding the market segments can take you far.

### The group leader perspective

**Fig. 3:**
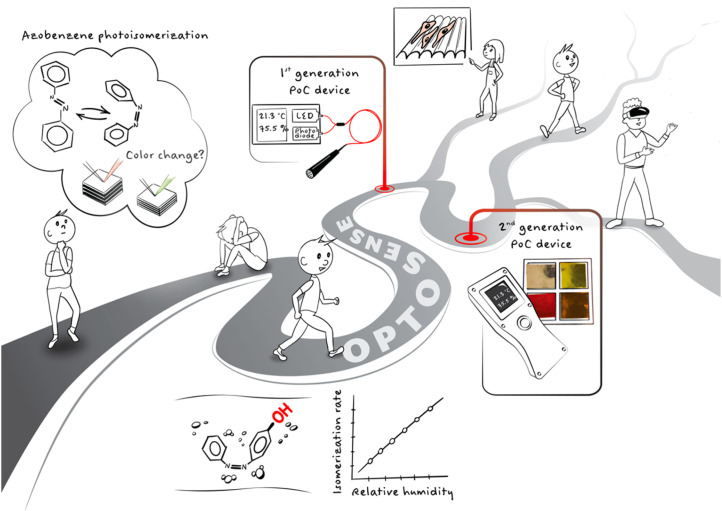
The OPTOSENSE journey. It started from failed experiments which, after thorough studies on the photochemistry of hydroxyazobenzenes in polymer thin films, turned into commercialization efforts and two generations of proof-of-concept devices. The journey continues and has also sparked other commercialization efforts in our group related to dynamic cell culture platforms and reconfigurable optical elements for augmented/mixed reality technologies.

OPTOSENSE has been quite a journey, and its main milestones are schematized in Fig. 3. Its project outcomes cannot be evaluated with the metrics (publications, citations) we are used to in the academic world. That said, its eventual goal – commercial activity – did not succeed, at least not yet. Despite yielding neither tens of publications/hundreds of citations nor a spin-off company, I consider the project a success, and am proud of its outcomes. It has been our first trial towards research commercialization, an endeavour that has left a permanent mark on the research done in our group through the broadened perspective on how we approach fundamental research.

For the persons involved in its different stages, OPTOSENSE has offered an excellent training ground, preparing them for life after academia. Mikko turned a poorly defined research idea into a new research concept. This was enabled by digging deep enough, and by not looking away when problems arose. Matti turned the research concept into a functioning device. The experience of product development, combined with project management and company outreach, provided him a confidence boost and skillset that a wholly academic setting could not have offered. Many other group members have now followed his lead and target deep-tech commercialization. The funding to support their efforts follows similar pathways as outlined above, starting from ERC PoC and continuing with BF R2B projects. And for our next two commercialization efforts – dynamic cell culture platforms and reconfigurable optical elements for augmented/mixed reality technologies, our faithful companion – azobenzene – is there to serve.

Finally, I would like to give three tips to those interested in commercializing blue skies research. First, **use help**. For writing the project proposal, the services of consultant companies can be a worthy investment that saves time. For IPR strategies and general coaching, university innovation services can be invaluable. Second, **designate an operational leader for your project.** Instead of trying to manage the project yourself, give space for other group members to grow. Being fully committed, they probably do it better than you anyway. Third, **go for it!** Do you think that research commercialization is not for you? That’s even more of a reason to give it a try and do things differently than what you are used to. Good luck!

## An early career perspective – treading the boundary between industry and academia

(Jennifer R. Hiscock)

When I was a PhD student, I had my heart set on making a career for myself within the defence science sector, what I was not focused on was a career in academia. However, after several failed industry-based job interviews, in which my feedback was always ‘you are too much of an academic, you should pursue a career in the University sector’, I wrote a fellowship proposal, which saw me move to the University of Kent (UK) as the Caldin Research Fellow in 2015 and promoted to Professor of Supramolecular Chemistry in 2022.

In hindsight, I must admit that the feedback from those early interviews, although hard to swallow at the time was correct. The University system has provided me with the creative freedom, support, and infrastructure to follow my dreams, to be an inventor and to focus on learning how to translate my innovations into the commercial sector. Since the first day of my independent career, I have focused my efforts on translational innovation.

One of the first bits of advice anyone will ever give you when you begin to undertake the translation of any scientific innovation is to focus on the commercialisation of one innovation at a time. However, I have ended up with two patent protected innovations both of which I am actively working to commercialise simultaneously, so I have already broken rule one, which may not be a sensible idea – only time will tell! The first of these innovations we refer to as the Supramolecular Self-associating Amphiphile (SSA) technology, and the second – Talin Shock Absorbing Materials (TSAMs).

### Number one: supramolecular self-associating amphiphiles (SSAs)

**Fig. 4:**
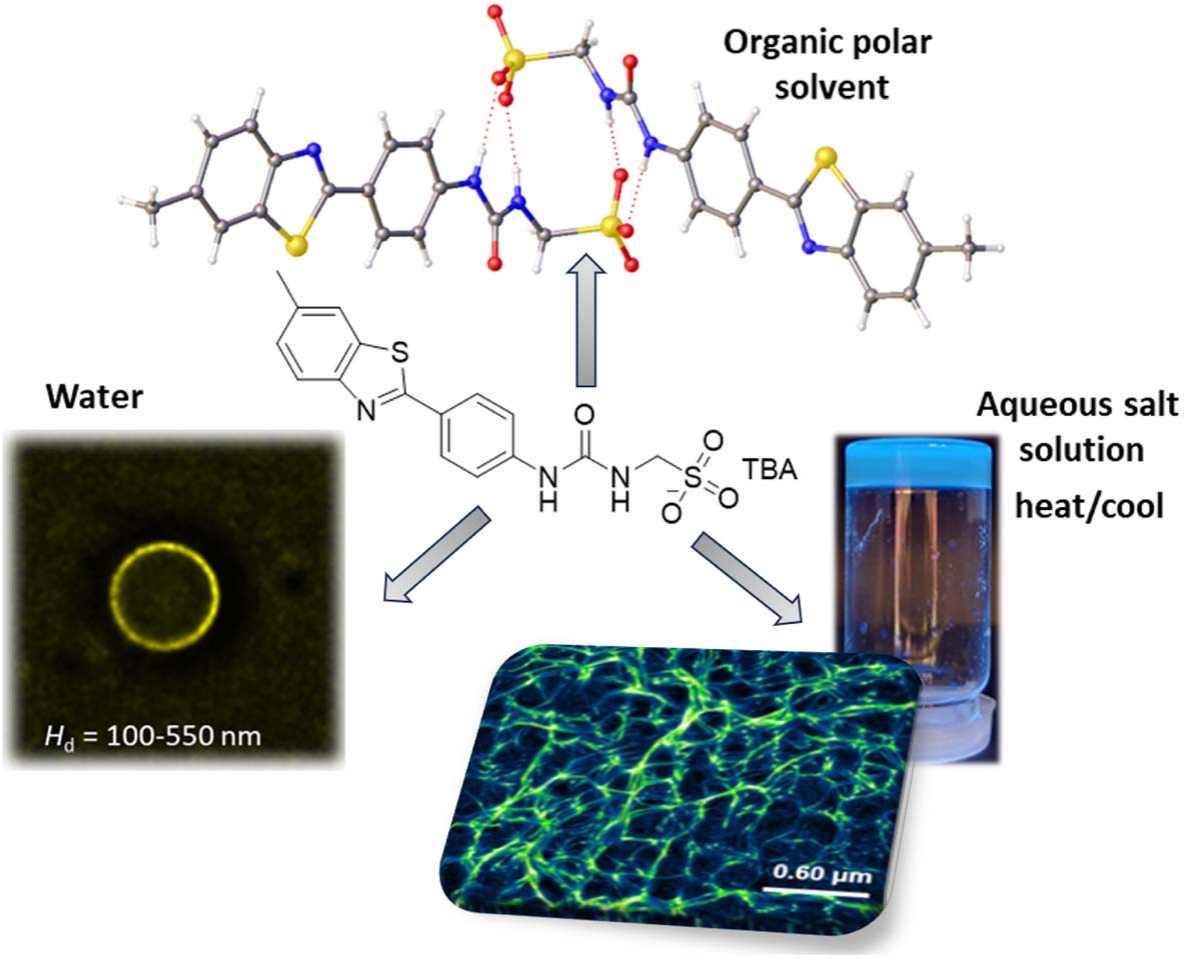
An example SSA, TBA = tetrabutylammonium. This SSA is intrinsically fluorescent due the presence of a benzothiazole functionality. In polar organic solvents, such as DMSO, we have evidence to support the formation of anionic, hydrogen bonded dimers, such as those identified in the solid state through single crystal X-ray diffraction studies. In water, this same SSA is shown to self-associate, this time producing spherical aggregates such as those identified through solution state fluorescence microscopy. However, upon the addition of salts, such as NaCl, followed by an annealing process, these spherical aggregates morph into fibres (also characterised through solution state fluorescence microscopy), resulting in the formation of hydrogels, with minimum gelation concentrations as low as 1.5 mg mL^−1^.

SSAs are a class of low molecular weight amphiphilic salts and related compounds, with structures related to that shown in Fig. 4. To date SSAs have demonstrated a broad array of therapeutic applications, having been shown to: (i) act as triggerable functional materials, as exemplified in Fig. 4;^17–19^ (ii) exhibit antimicrobial activity against model Gram-positive MRSA and Gram-negative *Escherichia coli*;^20^ (iii) increase or activate the activity of known antibiotic/antimicrobial agents against planktonic bacteria, including the ESKAPE pathogen *Pseudomonas aeruginosa*;^21,22^ (iv) demonstrate a ‘druggable’ profile when administered intravenously to mice;^23^ (v) have the potential to, or can act as drug delivery vehicles, dependent on material form ^17,24^

### Number two: talin shock absorbing materials (TSAMs)

The protein talin was first discovered in 1983 by Burridge and Connell.^25^ Talin is the protein that links the extracellular integrin protein receptors, found in animal cells, to the intracellular actin skeleton (the cell’s force-generation machinery).^26,27^ However, what is more exciting to us, is that this protein also acts as the cell’s shock absorber. ^28^

**Fig. 5:**
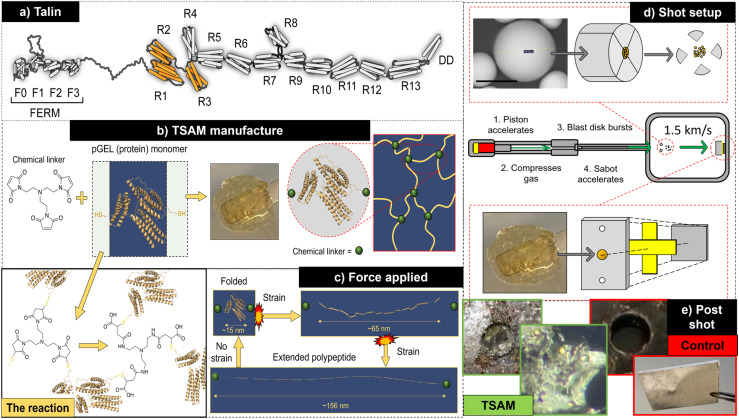
(a) A cartoon depicting the structure of the talin, including the R1–R13 domains which give this protein its shock absorbing properties. The R1–R3 domains used to produce the TSAM protein monomer have been highlighted in orange. (b) The structure of, and reaction between, the TSAM protein monomer and the synthetic tripodal linker used to produce the TSAM itself. (c) Cartoon illustrating the hypothesised response of the TSAM monomeric units, contained within the TSAM hydrogel, upon the application of force. (d) Experimental setup used to test the capability of the TSAM to absorb the impact of projectiles (basaltic particles) shot at 1.5 km s^−1^ into a 5 mm depth of TSAM (200 mg per mL protein monomer). (e) Images of TSAM material post shot. Here the material is intact and the spherical basaltic particles that have impacted the TSAM can be seen incapsulated within the material. Images of a control experiment in which a commercially available polyvinylpyrrolidone hydrogel has been destroyed under analogous shot conditions, and damage to a metal back plate incurred due to the failure of the control material.

Talin contains 13-rod domains that reversibly unfold upon the application of force (Fig. 5a).^28^ Within the scope of this innovation, we engineered the first three of these rod-domains into a protein monomer that was then cross-linked to produce a hydrogel, known as a TSAM (Fig. 5b and c).^29^ The thing that makes this hydrogel unique, is that the TSAM is able to retain the shock absorbing properties of talin itself. Furthermore, we also discovered that not only was this material able to absorb the impact of projectiles shot at supersonic speeds of 1.5 km s^−1^ (Fig. 5d), but it is also able to preserve the projectiles themselves, outperforming the industrial standard – aerogel (Fig. 5e).

### Where are we now?

I have filed six patents through the University of Kent concerning both innovations and have undertaken extensive patent searches, supported by an international patent expert, which has involved reading >5000 patent filings myself so that I truly understand the innovation landscape. We also have alerts set so that we actively monitor the patent landscape. These alerts are used to inform our ongoing patent strategy. While the fundamental aspects of both innovations are still funded through application to traditional academic research schemes, for the more translational research we are approaching venture capitalists, negotiating university spin out options and building experienced teams of advisors, and industrial experts to enable translation into the commercial space.

### What are the lessons learned to date?

At the present time, we believe both the SSA and TSAM innovations show commercial potential. We have climbed the first few technology development hills successfully, but now we have a huge translational mountain to climb. So, at this point in my journey, here are some of the lessons that I have had to learn, through feedback from mentors, learning from the success/failures of others, feedback from innovation pitches and acknowledgment of my own personal limitations.

#### Lesson 1 – Know your market

At the end of the day, your innovation will have to meet a need for which there is no current solution or, overcome a specific limitation inherent to an innovation that has already undergone successful commercialisation. In addition, your innovation will have to become something that an end user will want to use and, achieve all of this at a price point that your target market will be willing to pay. It is likely that your technology will have a direct or indirect competitor therefore, you must also understand how these competing innovations will affect the successful translation of your own innovation.

#### Lesson 2 – Learn when to pivot or start again

We all only have limited time and energy. It is our choice where to place our resources to best ensure success. It might be that you have used a lot of your resources to develop a single molecule/material, but this does not mean that molecule/material will achieve success. Having the confidence to walk away, rebalancing the outlay of resources and pivoting my approach has saved me more times than I can count.

#### Lesson 3 – Be resilient

This does not get highlighted enough, but for every success, there will be countless failures and for innovations that have become successful, there will have been countless people that have said ‘that idea will never work’. Failure of an innovation to translate is not the personal failure of the original inventor. What enables success, is the ability to pick yourself up and try again, or to push through when you truly believe an innovation has potential.

#### Lesson 4 – You do not have to be good at everything

I am dyslexic and this has made some aspects of academic study easy for me, and some others almost impossible. Therefore, I surround myself with individuals and collaborators that are complementary to myself, not only in scientific specialism, but in natural skill set – this way we create a mutually supportive environment where we can all achieve bigger and better things. To date, my commercial experience is limited, so I have coopted a team of industrial mentors with the experience necessary to guide effective technology translation within the relevant sector.

#### Lesson 5 – Build a supportive team

Innovation and translation are not a one-person journey, it is a team effort, so pick your team carefully and realise that it is ok for your team to change over time. You do not have to take the first offer that comes along. Do what is right for you and your innovation.

#### Lesson 6 – You are unique, no journey is the same

We are all unique, with a unique set of personal experiences, operating in a unique environment, therefore our journeys will all be different. Taking inspiration from others is fantastic but trying to mimic someone else’s journey is dangerous.

In summary, I feel that I am at the very beginning of my translational journey. The commercialisation of my innovations may never be successful, but life should make you happy. At present, although no journey is easy, I am enjoying this one, sharing what I am learning with others and undergoing a step-change in my own personal growth. I hope to look back on this article in 5–10 years to remind myself where everything started.

## Translating supramolecular chemistry: how a bad day in the lab led to SupraSensor Technologies

(Calden N. Carroll, Michael M. Haley and Darren W. Johnson)

**Fig. 6:**
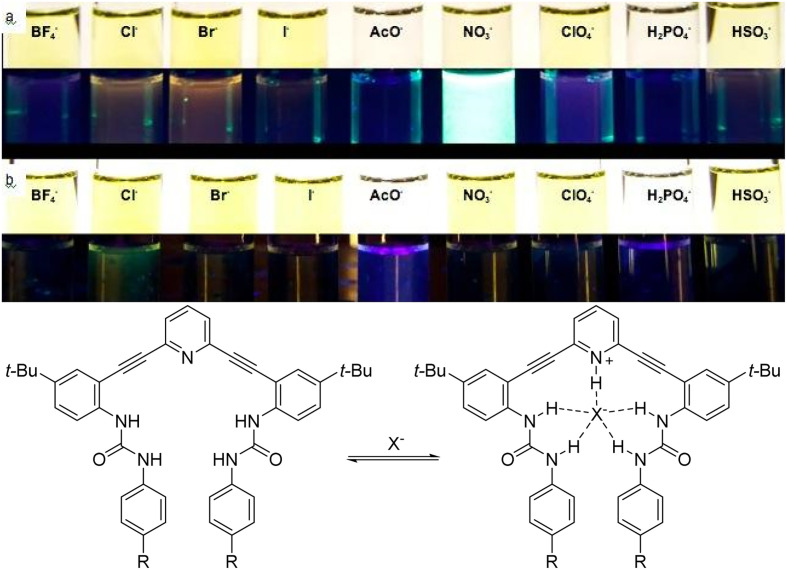
(Top) Vials containing two different arylethynyl bisurea anion receptors screened for their fluorescent response to anions; (bottom) hydrogen bonding receptors showing fluorescent response to various anions.

The story of SupraSensor Technologies starts with a bad day in the lab. Calden was a senior student finishing his last year of graduate school and considering options in industrial R&D or an academic post-doctoral position. The key experiment for his project had just been set up – we had pieced together knowledge from the literature, computational models, and empirical evidence from other graduate students’ work to generate a promising family of compounds for turn-on fluorescence sensing of chloride using our arylethynyl receptor scaffold. Once these new candidate receptors were prepared, a quick “stained glass window” experiment using cuvettes filled with solutions of various anions did not require a well-trained eye to recognize that the top receptor scored a hit (Fig. 6), but not for the right anion!

Concurrently, our collaborator John Naleway at Marker Gene Technologies was experimenting with these receptors as fluorescent indicators for chloride ion channel studies. As it turns out, fluorescence imaging for chloride ion channel experiments does not work well with a receptor that makes the background nitrate buffer fluoresce too. As any experienced graduate student would know, bad news is a good time to take a break, so Calden went fishing, which is perhaps a suitable metaphor. Our modular approach to developing receptors for selective response to anions is akin to fishing; if you can properly “match the hatch” you can select for the trout you want in a river full of whitefish.

To round out these early results, we published a communication about the turn-on/turn-off fluorescence observation and took on the lengthier task of writing a review article with John on the use of this class of receptors in cellular imaging, which also became the introductory chapter to Calden’s dissertation.^30,31^ A chloride selective receptor was eventually identified, and Calden successfully defended his thesis in November 2011. At his defence and in response to Calden’s joke about fishing instead of re-doubling his efforts in the lab, one of his committee members innocently asked a question to the effect: “But isn’t there a need for nitrate sensors worldwide? What are you doing after graduation?”

### National Science Foundation Innovation Corps

In spite of the joke, we were now seriously entertaining the possibility of a startup company, but how exactly does one start up a startup? We put together a team of Mike, Calden, Darren, Prof. Bruce Branchaud (an emeritus faculty member who was pursuing an industrial career at Invitrogen Corp), as well as Augie Sick, our key business mentor who had worked on mergers and acquisitions for Invitrogen for many years.

At one of our first team meetings (Jan 11th, 2012), Bruce mentioned that he had recently seen an announcement for a new NSF program for startup teams called Innovation Corps (I-Corps). Basically, I-Corps would fund a group for US$50 000 for six months to determine whether research with an NSF-lineage was a “go” or “no go” for launching a startup. Fortunately, Calden was funded by Mike’s NSF grant during his first two years in graduate school when the initial nitrate discovery was made, so we emailed the NSF with our nitrate sensing idea.

In the 30 days between January 11th and February 10th, 2012, we submitted a white paper, were invited to a 15-minute phone interview, and experienced the most unusual call we’ve ever had with NSF program managers. As soon as they came online, all they asked about was the technology, the business model, and so on. They never asked about the science, except to put Calden on the spot to ask him, “What would you do with $50 000?” Calden’s quick response, and the team’s disclosure of an issued patent led to a palpable and prolonged silence (surprise possibly?) on their end.^32^ After 10–15 seconds, the lead PM finally said that he was pleased to hear this and that they would get back to us soon, and with that the call was over. We all looked at one another in a bit of disbelief. Eight days later proved the call definitely went well as we received an email inviting us to submit a 5-page proposal – due in 4 days! We quickly assembled the proposal, the required NSF supporting documents, and hit the submit button the afternoon of February 22nd. Much to our surprise, we received the award notification before noon the very next day! With seed funding secured, it was now time to see if we should start up the startup.

**Fig. 7:**
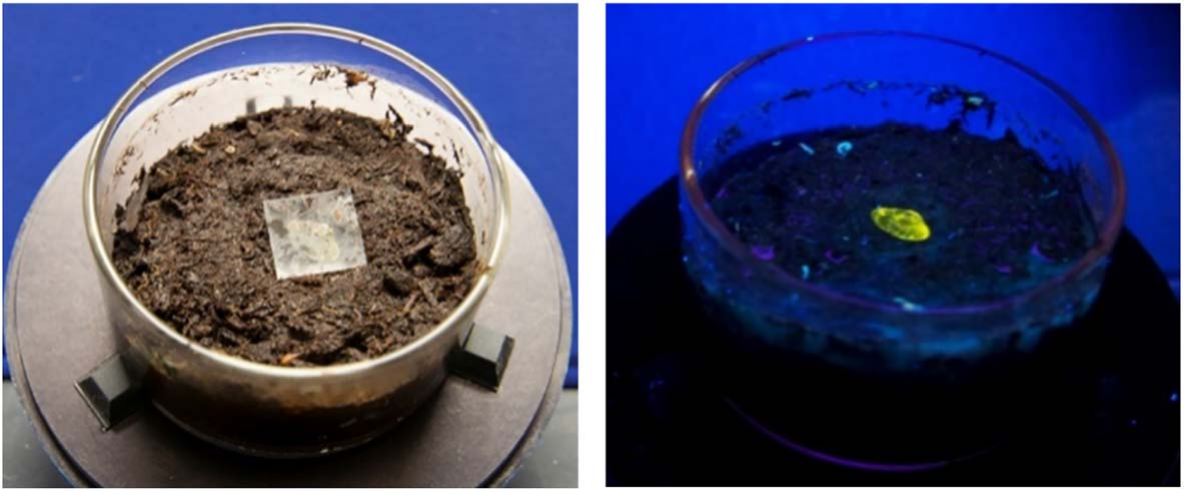
Polymer film containing a small amount of the nitrate-sensing receptor molecule under normal (left) and UV (right) light.

NSF I-Corps focuses on customer discovery and wants to get the scientists and engineers “out of the lab and building” to talk to 100+ potential customers of their technology. At the time, we did not even know what end user market our most likely customers would come from. Calden made the wise decision to focus on the interviews and wait to iterate the science until he had more market data. The only experiment he performed during the I-Corps programming was to show that the fluorescent response in the test tube was maintained in a polymer film that could identify nitrate-rich soil (Fig. 7). Calden completed the I-Corps project with a reasonable business model canvas, a stronger sense of the market needs in sensing nitrate in precision agriculture, a company name (SupraSensor Technologies, SST), and a lot of momentum: he was awarded the Best in Show prize for his final presentation, and the NSF Deputy Director congratulated him afterwards with the encouragement that we should submit a Small Business Innovation Research (SBIR) grant for the next grant cycle … due in 6 weeks.

### A timely sabbatical request

Darren was up for sabbatical from the University of Oregon (UO) in Fall 2012. As part of his sabbatical request, he wrote that our team was in the process of launching a startup, and that he would use his sabbatical to help launch the venture, apply for State and Federal grants, assist in developing the company’s management structure, and help develop the technology and market awareness. Our small technology transfer office was instrumental in helping us build SST’s IP portfolio, including the already issued patent. The UO deserves a ton of credit for taking a risk on early-stage discoveries from 2008 and before that we had no real clue of what might have value at the time.^33^ Our technology transfer office (now called “Innovation Partnerships”) continues to work proactively with faculty to secure early-stage IP, build our pipeline of innovation activities, and accelerate the culture change in research translation at UO.

The sabbatical was a tremendous time of growth for Darren. He knew nothing about launching a startup beyond the limited training from I-Corps, but he could help with grantsmanship (not that Calden needed much help, but the extra pair of hands were probably useful to give Calden more time for other activities). In that short period of time the team produced many successful proposals,^34^ raised some funds from the five founders, formed the company, and set up the initial corporate structure; fortunately, none of the mistakes we made in that flurry of activity proved fatal …

### Forming and growing a small business in Oregon

The traction we received during our I-Corps experience meant two things. Firstly, we had a short deadline to submit a small business proposal … for a business that did not yet exist. Calden dug in and formed an Oregon-based limited liability corporation with no previous experience, and no understanding of the difference between an “agent” of an organization and a “member”. The first draft of the operating agreement for SupraSensor Technologies, LLC was formed without Calden as a member of the company, but at least was formed in time to start the lengthy and opaque process of registering for the series of certifications that made a small business capable of applying for federal funds in the U.S. A SAM number, DUNS number and amendments to the structure of the LLC all became minutiae that threatened to undermine a timely submission of our first NSF SBIR grant.

One of the first challenges came from the “burden of choice”. Our naïve understanding of customer discovery suddenly brought the number of novel technologies coming out of Darren and Mike’s labs all at once crashing into the arena of possible solutions applicable to a growing number of markets. To a curious chemist’s mind, the prospect of exploring the fit and size of each of these application spaces was both exciting and overwhelming. We envisioned a corporation that was laser focused on identifying and assessing not just the supramolecular chemistries that were emerging from our university but providing those services to friends and collaborators within our field. That sort of business demanded much more flexibility in how it handled in- and out-licensing, attribution and equity, and speed of operation. It became increasingly clear that our simple LLC structure was insufficient for the scope of the business we had in mind. For this we leaned on our legal counsel, and the depth of experience of our business mentor and co-founder, Augie Sick. We decided on a simple umbrella structure, with a parent corporation and board that retained intellectual property rights, which would be sublicensed to subsidiary businesses focused on each of the markets we had envisioned for our technologies. From our earliest customer discovery work, we knew we had a compelling first story to tell at the food–energy–water nexus, but no money.

Federal grant funding cycles can be slow, especially compared to the speed with which a small team needs to move. State grants can be significantly faster, and those coming from centres of excellence or innovation initiatives can be faster still. This does not mean that the colour of that non-dilutive money is always the same, though.^34^ Budgets and allowable expenses, especially those for legal work (whether IP or operational), are difficult to align. The opportunity cost of this type of funding structure is an important part of the business plan of any small university spin out, as we discovered. The hidden benefit of those guardrails on budgets is that it required the team to stay focused on the beachhead we had identified and was likely a huge boon in steering us away from tumbling down the rabbit holes of these other possible markets and products.

### Further leveraging of university and community resources

We were lucky enough to make our first hire out of the Master’s Internship Program run out of the Materials Science Institute at the University of Oregon.^35^ We returned to that well a few more times—it cannot be understated how helpful it is to have engaged, excited student entrepreneurs involved at all stages of start-up development. They provided fresh eyes on the technical problems in the lab, more concise and accurate translations of new data into readable proposal sections, and bonkers (but viable!) new ideas during late night brainstorming sessions. The technical team at SST would ultimately include 7 masters or doctoral student scientists from UO.^36^

### The rubber meets the road

At this point in our journey, we had developed working chemical sensors, had designed supporting systems with size, weight, power, and cost requirements defined by our customer discovery processes, and a vision for how the minimum viable product would operate and look. What we needed were the interfacial skills: firmware and app development, design for manufacturability, and branding and recognition. We were lucky to get a lot of free press through our transparency about how we had stumbled ourselves into a promising start-up venture.^37^ Our elevator pitch for precision agriculture was simple: we reduced time-to-data for mission critical, weather dependent, and expensive fertilization operations in broadacre crops. This had the bonus of reducing the impact of overapplication of chemical fertilizers that led to degradation of our water resources, while wasting a significant amount of money, energy, and embodied carbon through the nitrogen fixing Haber–Bosch process.

Our initial prototype was a chemical field effect transistor (ChemFET), functionalized with a proprietary membrane including a supramolecular receptor for nitrate that resulted in the selectivity, sensitivity, and robustness required for use directly in field soils. Our grant-funded proof of concept, our in-lab testing in controlled soil environments, and a single field mesocosm trial with samples from a large partner farm in eastern Oregon gave us confidence that we were capable of meeting the accuracy requirements for laboratory soil testing in field. This would mean a significant reduction in time-to-data and addressed one of the key concerns of the potential customers we had talked to: early season decisions about fertilization in agriculture are often made during transient weather windows, but require an offsite laboratory to process and analyse the soil samples from each field. The resulting backlog of samples at local labs meant further increased wait times, and led to most growers significantly overapplying fertilizer to be certain they could meet their yield requirements prior to obtaining the necessary measurements from each field.

### Our first pivot

The soil nitrogen cycle is a complex, multifaceted system with both abiotic and biotic drivers that can vary dramatically even within a single field. Fertility prescriptions focus on nitrogen rather than nitrate (to account for all sources of nitrogen that may be present) and so fertility decisions are made on the basis of “nitrate-N in bulk soil”, not nitrate concentration in the water therein. This seemingly slight change in nomenclature had significant implications for the actionability of the data we provided. Our first customer-led mesocosm studies were a disaster for actionability. The soil scientists loved the actual data, the growers (and customers) had no idea what to do with it, since it was not in units of “nitrate-N”. Everyone had been talking about what they would do with “N data” or “nitrate data” interchangeably, and no one had asked the question about the real-world implications for this slight change in referencing the concentration of the molecule or of the atom.

### Following the exit signs

The reality of our path to market became clear: we were not creating a simple sensor company, but a data-driven decision support company that required a substantial amount of field data, agronomic expertise, and multiple years of proven in-field results. We had some interest from laboratories engaged in soil testing, from agricultural consulting firms, from fertilizer companies, and from “big data” players. Our partnership pipeline for field testing included many of these, with one of them already being an established, billion-dollar company engaged in agricultural data products. We entered into a simple research agreement and sent some prototypes “over the fence” to be blind tested against laboratory protocols. We passed with flying colours.

The success of our external testing led to further field trials with partners, and increased interest in SST’s technology to meet an already well-defined data need for the agtech products that these partners were actively developing. On SupraSensor’s side, the board met and discussed potential options: licensing, recurring sales models, or a hard break and pursuit of our own opportunity as a standalone agtech data provider.

The structure of our organization lent itself to licensing: we had intended to hold all core intellectual property at the parent corporation (SupraSensor Enterprises, Inc) and sublicense to the subsidiaries (in this case SupraSensor Technologies, LLC) for each major market segment. In addition, there were other candidate supramolecular technologies that had promising applications in other large market segments that we wanted to pursue, and licensing would then provide us with the breathing room to pursue other markets with adjacent technologies still held by the labs at UO. While a sound plan, our rush to form the company, our retroactive incorporation to attempt to execute on this plan, our failure to properly structure our intellectual property licenses, and our relative inexperience with business had created a bit of a mess that did not align as easily with the plan on paper.

It became increasingly clear that licensing was the crispest way to both demonstrate a successful exit and prove the concept of a supramolecular platform focused business structure. Our initial negotiations included two partner businesses as sole licensees, but was relatively quickly reduced to one as we narrowed down on the strengths of our licensee’s business models. Ultimately, the acquiring company voted to acquire the team and technology at SST LLC in both a talent and technology acquisition in the field of nitrate sensing in agriculture. An AgFunder News article provides additional context for readers interested in hearing Calden’s thoughts ∼9 months after the acquisition.^38^

### Future outlook

The acquisition led to many new opportunities for the strong team at SST, however it effectively ended the relationship between the UO research team and SST. At the same time, it provided many new fundamental science research directions for the UO team. Academic entrepreneurship is certainly on the rise, and more and more universities are supporting these efforts in substantial ways to invest in the skills of their teams and provide financial support through non-dilutive grants. In Darren’s case, a sabbatical was a key to his involvement in SST, which suggests that on-demand sabbaticals are a low-cost way for universities to support innovation activities. There is still a pressing need for low-cost, selective, deployable sensors for environmental and biological applications, so the opportunities for supramolecular chemistry are still very strong in the next 5–10 years. We joke about “getting the band back together”; maybe we should get more serious about that …

## Launching INNOVOTEX: the next generation of texaphyrin drug conjugates (TDCs)

(Jonathan F. Arambula, Krystle Karoscik and Jonathan L. Sessler)

**Fig. 8:**
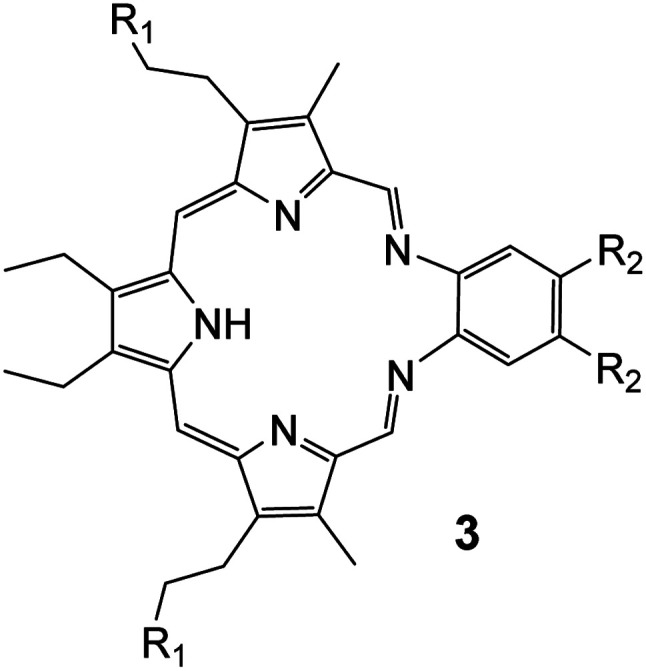
Structure of the free-base form of texaphyrin. It acts as a monoanionic ligand and is stabilized through metal cation complexation. In the bulk of the studies referred to in this section, the metal cation is Gd(iii).

Porphyrins are the archetypal bioinorganic ligand, being found in a range of metalloenzymes. These macrocycles have inspired research across the chemical sciences from catalysis through to photovoltaics. In 1988 we reported the first class of macrocycles known as texaphyrins (3) (Fig. 8), an expanded analogue of the classic porphyrin skeleton.^39^ Our thoughts immediately turned to their potential medicinal chemistry applications. Porphyrins are known to accumulate in cancerous tissue and our hypothesis was that texaphyrins might do the same. This, combined with their ability to coordinate larger metal ions than accommodated by their smaller porphyrin analogues, including medically relevant cations, such as Gd(iii), and their flexible redox chemistry, which allows them to mediate the production of reactive oxygen species (ROS), led us to develop texaphyrins as potential chemotherapeutic agents.

### Pharmacyclics: a first push to get texaphyrins into the clinic

Pharmacyclics, Inc. was co-founded in 1991 by Richard A. Miller, MD, who served as its first President and CEO and one of the authors (Sessler) to focus on the translation of texaphyrins to the clinic. Two water-solubilized versions, known as motexafin gadolinium (MGd) and motexafin lutetium (MLu), were taken into clinical trials as the result of Pharmacyclics’ efforts. The initial thinking was that MGd could be used for magnetic resonance imaging (MRI) of tumours, as well as redox cycling-based therapy, whereas MLu, would serve as a sensitizer for photodynamic therapy (PDT – the destruction of cancer cells *via* light-initiated ROS). Over time, MGd was selected as the lead and taken on to Phase III trials as an adjuvant for the radiation-based treatment of brain tumours arising from metastatic lung cancer. The final Phase III trial did not meet its primary endpoint of delaying neurological progression. At least one post-mortem analysis was been carried out.^40^ A consensus view at the time, and recapitulated in the ex post facto analysis, was that not meeting the trial primary endpoint was in large measure due to 2 out of 64 clinical sites waiting almost two months to treat their patients. It was observed that the 2-week randomization at North American sites resulted in meeting the Phase III endpoint, but unfortunately, the delayed randomization at the European sites did not produce such a response.^41^ It should also be noted that an adaptive trial design was not implemented in the Phase III trial. There was thus some thought that FDA approval might still be forthcoming for MGd.

However, by 2007, it was clear that was not to be. Thereafter, all intellectual property encompassing these macrocycles was transferred to The University of Texas at Austin (UT Austin). Wisely, under Dr Miller’s guidance, Pharmacyclics had already begun to focus on two backup programs in-licensed from Celera, Inc. One of these, a covalent Burkett tyrosine kinase (BTK) inhibitor, showed tremendous promise. By 2013, it had become the FDA-approved drug Imbruvica (ibrutinib). The commercial potential of Imbruvica led to the acquisition of Pharmacyclics, Inc. by AbbVie for $21B in the spring of 2015.^42^ While Dr Sessler and Miller were no longer associated with Pharmacyclics, Inc. by that juncture, it should be emphasized that the key scientific advance embodied in ibrutinib, namely the use of a covalent strategy to slow the clearance of drugs and thus increase their efficacy, was recognized while they and other researchers were still involved with Pharmacyclics. The success of ibrutinib helped popularize the concept of covalent drugs. It also made BTK attractive as a “druggable” target. As a result, Imbruvica is facing increasing competition in the market. Nevertheless, it enjoyed roughly $0.9B in sales for the 4th quarter of 2023.^43^

### The texaphyrin journey: from Pharmacyclics to INNOVOTEX

As Pharmacyclics’s new focus was on ibrutinib, we were free to relaunch texaphyrins as macrocycle-based cancer-localizing drug carriers. The tumour-localizing features of the texaphyrins, which paralleled what had been seen for many porphyrin and porphyrin analogue PDT photosensitizers, were noted early on in animal studies and subsequently confirmed in the clinical studies of MGd as reflected in both MRI scans and patient biopsies. For instance, in the case of glioblastoma multiforme, a difference in concentration between diseased and surrounding tissues of approximately 70 : 1 was seen.^44^ This attribute, coupled with the relatively low toxicity of MGd (on a cancer therapy scale), led to the consideration that MGd and its congeners could be used as macrocycle-based carriers to bring relatively toxic and non-specific oncology drugs to tumour sites with greater fidelity than could otherwise be achieved. In our view, this approach bears an analogy to that of antibody–drug conjugates (ADCs). Discussions with Dr Zahid Siddik of the MD Anderson Cancer Centre (MDACC) led to the suggestion that this strategy would be particularly advantageous in the case of platinum drugs. In particular, Dr Siddik considered that a roughly 50% increase in the area under the curve for platinum at the tumour, without an increase in toxicity, would translate into a potential doubling in the five-year survival rate for ovarian cancer.^45^

The testable hypothesis was that this could be achieved through effective texaphyrin-mediated localization. It was also thought likely that the use of a texaphyrin core might change the clearance of platinum drugs away from the kidneys towards the liver, thus reducing the nephrotoxicity that is often dose-limiting in the case of cisplatin. Arambula joined the Siddik–Sessler team in 2008 as a joint MDACC-UT Austin American Cancer Society postdoctoral fellow to begin testing these ideas. Although the jury is still out, synthetic advances have been made that led to the development of OxaliTEX (5) as a lead texaphyrin platinum(iv) prodrug in the Sessler lab at UT Austin. Biological testing of this so-called texaphyrin drug conjugate (TDC) in mouse models has also been carried out at MDACC, UT Austin, and through contract research organizations (CROs).^46^ The promising results obtained with 5 led to the filing of new patent applications on its composition to protect the intellectual property (IP). These results also provided a key trigger for the founding of INNOVOTEX Inc by the authors in 2022, with the aim of taking OxaliTEX into the clinic. The creation of OxaliTEX and the progress of INNOVOTEX are described in the following sections.

### The creation of OxaliTEX

**Fig. 9:**
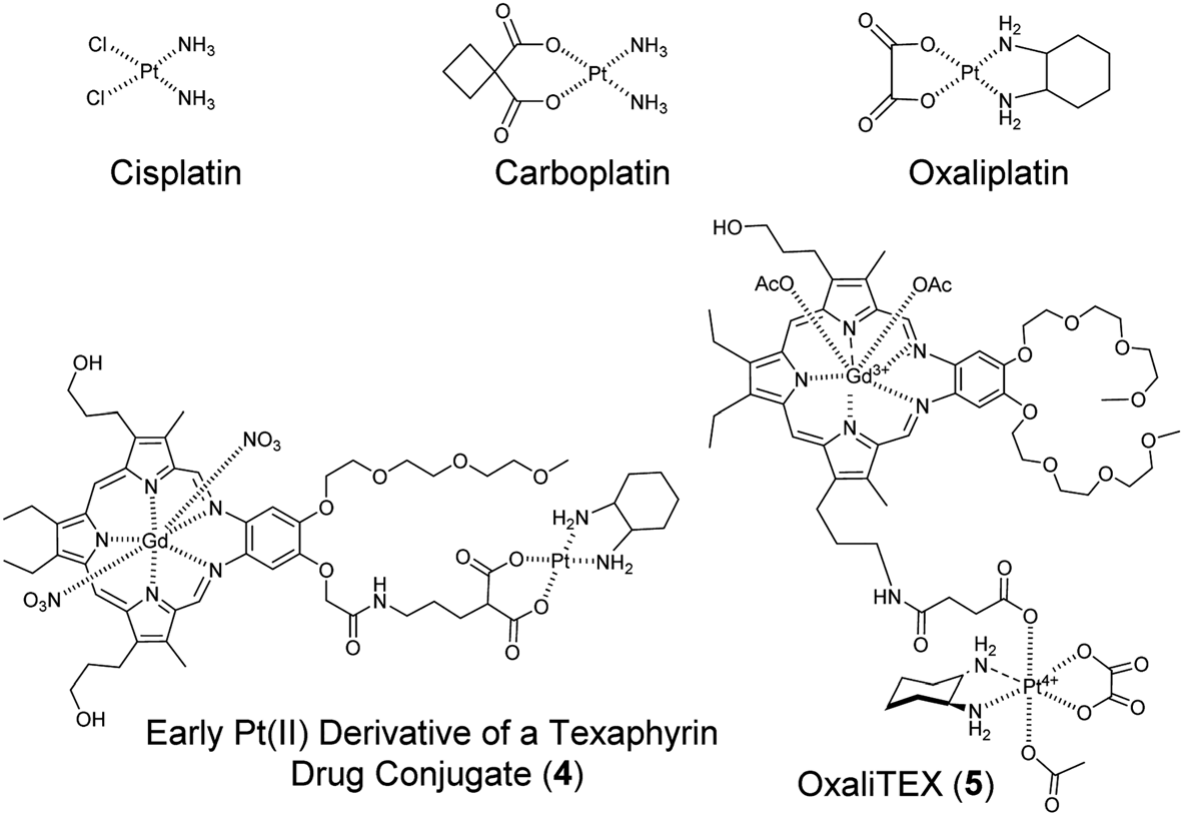
FDA-approved platinum agents cisplatin, carboplatin, and oxaliplatin. Also shown are the structures of two texaphyrin drug conjugates, namely 4 and OxaliTEX (5).

Early on, Arambula focused on creating texaphyrin–platinum conjugates that incorporated analogues of the FDA-approved platinum(ii) drugs cisplatin, carboplatin, and oxaliplatin (Fig. 9). These efforts had antecedents in early, largely unpublished efforts carried out at Pharmacyclics.^47^ Several iterations then led to the synthesis of a conjugate 4 containing a Pt(ii) centre tethered through a malonate chelating group.^48,49^ A key element in the design of conjugate 4, as opposed to earlier platinum conjugates, was the incorporation of the diaminocyclohexane ligand seen in oxaliplatin.^50^ Oxaliplatin, and presumably other platinum agents containing lipophilic ligands around platinum, targets the cancer ribosome and induces ribosomal biogenesis stress.^51^ This mode of action differs from the DNA damage response pathways triggered by cisplatin and carboplatin. The use of diaminocyclohexane as a ligand also produces species that can circumvent the cross-resistance profiles of cisplatin and carboplatin in wild type p53 (wt-p53) platinum-resistant ovarian cancers.^52^ There are a number of wt-p53 cancers that are resistant to cisplatin and carboplatin. This resistance is ascribed to the presence of several different epigenetic factors that prevent p53 activation with DNA damaging agents. Compound 4 and OxaliTEX (5) are able to reactivate wt-p53 in these cancers *via* the induction of ribosomal stress. We thus postulated that conjugate 4 might have particular utility in treating wt-p53 cancers that are resistant to traditional agents, such as cisplatin and carboplatin.

**Fig. 10:**
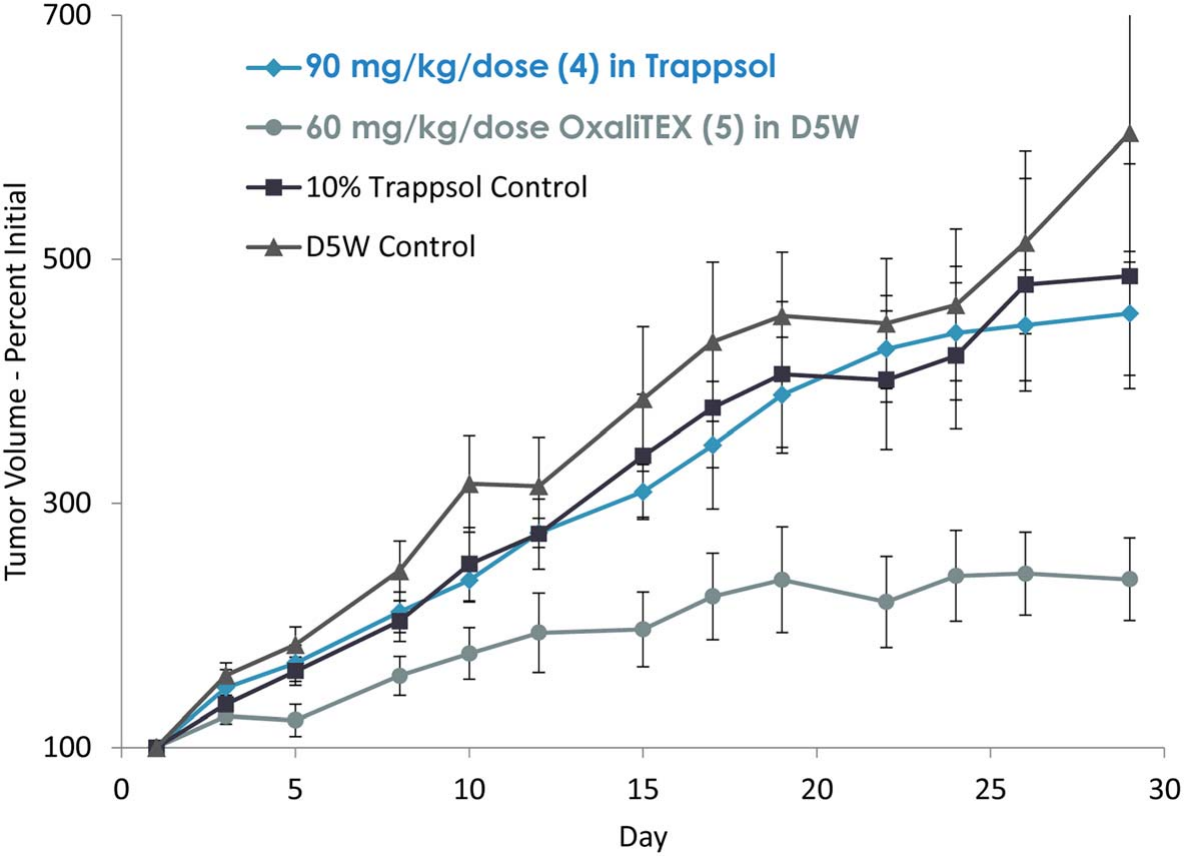
Efficacy study of A549 human lung cancer hind flank tumour xenografts in mice. The comparison involves the intravenous administration of four doses each of 4 and OxaliTEX (5) over two weeks. TDC 4 was administered at the maximum tolerated dose (MTD) and 5 was administered at a dose that was ∼85% of the MTD. Error bars represent the standard error.

Although good *in vitro* efficacy was seen with 4,^53^ difficulties were encountered in formulating it for *in vivo* studies. Moreover, the results from initial tumour regrowth studies were less than promising (Fig. 10). Thus, when Dr Gregory Thiabaud joined the team as a postdoctoral fellow, an effort was made to create a Pt(iv) texaphyrin conjugate. These synthetic efforts culminated in the preparation of OxaliTEX (5).^54^

A key design element underlying the choice of OxaliTEX (5) as a synthetic target was an appreciation that the platinum centre would be hexacoordinate in its tetravalent state but four-coordinate and square planar in its divalent state. The axial ligands around the Pt(iv) centre could thus be used to tether an MGd-derived texaphyrin core to the metal atom. Reduction to Pt(ii), expected in the reducing tumour microenvironment, would then serve to release an active drug form, specifically the FDA-approved agent oxaliplatin. The specific choice of OxaliTEX as a target was also guided by preliminary studies revealing that an acetate anion as the sixth ligand about the Pt(iv) centre provided a good balance between kinetic stability and release of oxaliplatin under reducing conditions. As with 4, OxaliTEX (5) incorporates the diaminocyclohexane ligand seen in oxaliplatin, meaning that it too would have presumed benefits in treating wt-p53 platinum-refractory or resistant solid tumours. This key inventive step, allowed for the filing of new composition of matter claims in multiple international jurisdictions thus supporting commercialization efforts.

To date, multiple mouse model studies have been carried out that lend credence to the above assumptions. An initial biodistribution study in mice revealed greater hepatic accumulation than seen for oxaliplatin. This led to considerations that it might display a lower level of toxicity than oxaliplatin due to anticipated cytochrome p450 metabolism of texaphyrins. In fact, the maximum tolerated dose (MTD) for OxaliTEX (5) on a per platinum mole basis was roughly 3 times that seen for oxaliplatin. In an initial study involving a subcutaneous xenograft mouse model of A549 lung cancer, improved outcomes were seen for 5 relative to 4 (Fig. 10).^53^

**Fig. 11:**
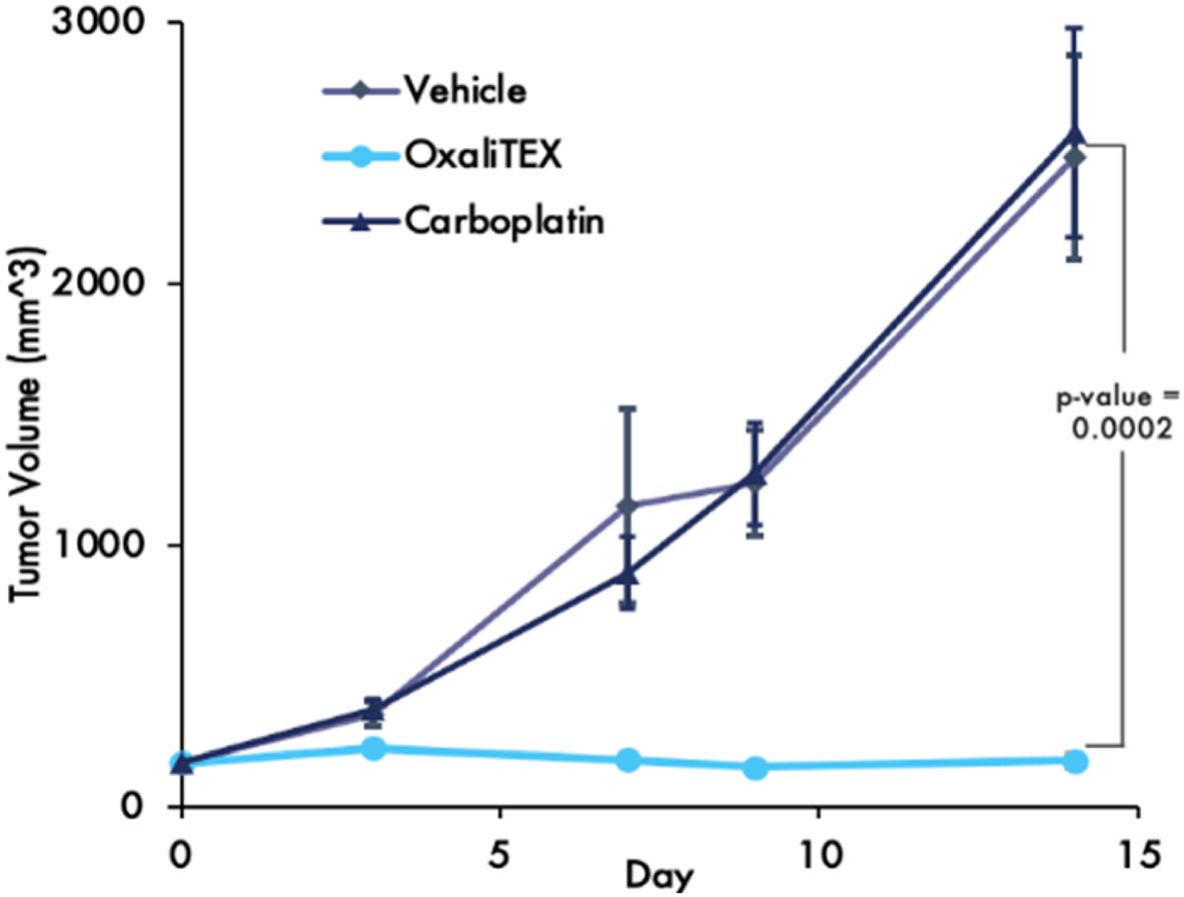
Treatment of a patient-derived xenograft (PDX) tumour of a platinum-resistant wt-p53 serous ovarian cancer in mice with clinicians’ choice carboplatin compared to OxaliTEX (5).

A particularly noteworthy study was carried out using a patient-derived xenograft of wt-p53 platinum-resistant ovarian cancer. In this study, both OxaliTEX (5) and carboplatin, used as a standard of care positive control, were administered at their respective MTDs. As can be seen in Fig. 11, neither vehicle nor carboplatin produced an appreciable benefit in terms of retarding tumour growth. Conversely, no apparent tumour growth was seen with OxaliTEX (5) throughout the two-week study.^46^ Little weight loss, a crude indicator of potential acute toxicity, was seen for 5 during these studies and in parallel tests involving cancer-free mice. OxaliTEX (5) was found to possess good aqueous solubility, making it easy to administer through i.v. injection. It thus showed the key attributes expected of a potential drug lead.

The findings obtained with OxaliTEX (5) were taken as an initial validation of the so-called TEX Core strategy wherein texaphyrins are used as tumour-localizing carriers for active anti-cancer agents. To develop this promise, in 2022, the authors co-founded INNOVOTEX Inc., a Delaware company headquartered in Austin, Texas. INNOVOTEX has as its mission the development of OxaliTEX (5) as an experimental drug. INNOVOTEX also seeks to showcase more broadly the promise inherent in the TEX Core approach to cancer drug development.

### Current efforts and future plans for INNOVOTEX Inc.

Building on the results obtained through 2022, INNOVOTEX is pursuing a biomarker-driven approach for the preclinical and, ultimately, clinical development of OxaliTEX (5). The target indications for this drug candidate are thus wt-p53 solid tumours, with consideration for those that are platinum-refractory or -resistant. In this context, INNOVOTEX expects clinical adoption for OxaliTEX (5) to evolve in due course from second- to front-line therapy for multiple indications.

In terms of business development, there are a number of reinforcing considerations that INNOVOTEX is using to position OxaliTEX for its putative future adaptation in the clinic. For example, INNOVOTEX is using market analyses to evaluate the dynamics of OxaliTEX (5) as a potential product and its target audience. Key components within this framework include (i) a thorough understanding of potential consumers, (ii) the ability to produce OxaliTEX (5) on scale, and (iii) convenience of use. These considerations for the therapeutic use of OxaliTEX (5), particularly, and the TEX Core platform, in general, are detailed below.

First, INNOVOTEX plans to implement biomarker profiling for the targeting of multiple solid tumors. One such indication of interest is ovarian cancer. Ovarian cancer is the number one cause of gynaecologic mortality,^55^ with an estimated 300 000 women diagnosed on an annual basis worldwide,^56^ of whom 80–90% will develop platinum resistance. The median survival rate for patients diagnosed with platinum-resistant ovarian cancer is 9–12 months,^57^ with fewer than 15% of patients responding to subsequent chemotherapy. These statistics highlight an urgent and unmet clinical need for new platinum-based therapeutic development, as embodied in INNOVOTEX’s lead OxaliTEX (5). A key to INNOVOTEX’s profiling is to target wt-p53 platinum-refractory or platinum-resistant solid tumours. Based on a rationally considered regulatory strategy, INNOVOTEX is prioritizing ovarian cancer as our initial indication to address an urgent and unmet clinical need for the treatment of rare disease subtypes that will allow us to leverage an expedited approval pathway under orphan drug designation. Additional indications, primarily colorectal and non-small cell lung cancers, demonstrate improved efficacy using OxaliTEX and will be considered as part of our clinical strategy.

Second, as part of its initial market analysis, INNOVOTEX identified a variety of other solid tumour indications that exhibit the same functional wt-p53 biomarker profile. The profile percentages are expected to allow potential scalability into other markets to be determined. This analysis is expected to de-risk further the use of OxaliTEX (5) in solid tumours, as well as inform better clinical trial strategies and potential clinical adoption opportunities.

Third, INNOVOTEX is focused on understanding the competitive landscape. Analyses carried out to date include a comprehensive overview of two sectors: (1) current ovarian cancer therapeutics and (2) platinum-resistant therapeutics. INNOVOTEX has identified therapies in all stages of development through commercialization. This has allowed a thorough understanding of therapeutic success and limitations for each modality, target market, clinical use, commercial strategy, and company infrastructure. In an initial assessment, several early-stage companies focused on developing ADCs and immunotherapies as potential competitors. However, the strategies pursued by these companies are plagued by significant challenges associated with tumour trafficking and migration, as well as immunosuppressive factors in the tumour microenvironment that can become problematic.

Based on the above analysis, the authors believe that INNOVOTEX is significantly differentiated from its possible competitors. From a modality perspective, the TEX Core strategy and INNOVOTEX’s lead, OxaliTEX (5), both involve a macrocycle drug conjugate. The texaphyrin core itself has been clinically validated to localize to solid tumours and was found to be well-tolerated (on a cancer therapeutic scale) in studies involving approximately 1000 patients. The idea of replacing an antibody with a small molecule with a track record for cancer targeting is innovative and, as importantly, represents a key market differentiator. Moreover, as noted above, INNOVOTEX’s initial target market is wt-p53 ovarian cancer subtypes, as opposed to the crowded market of high-grade serous ovarian cancer. INNOVOTEX is also targeting the *ca.* 30% of subtypes ovarian cancer that are *de novo* refractory cancers that are wt-p53. To our knowledge, no one else is targeting these cancer subtypes.

The market analysis revealed *inter alia* two later-stage ADC companies that consummated strategic industry partnership deals upwards of $1B yet failed to meet their primary endpoints. These failures underscore further the urgent and unmet need for differentiated therapeutic modalities within the platinum-resistant and ovarian cancer markets. INNOVOTEX believes that this need, in turn, will encourage established pharmaceutical companies to seek other novel assets for strategic partnerships.

Even as an early-stage company, INNOVOTEX believes that a robust clinical trial strategy is essential. The studies carried out to date are helping guide INNOVOTEX’s proposed clinical trial approach with the goal of creating data sets that will facilitate commercialization and clinical adoption. INNOVOTEX intends to implement early on an innovative, adaptive trial design to increase efficiency and augment chances for success. Importantly, this approach provides opportunities to streamline and optimize interim data outcomes and modify the trial design to support later-phase trials. Our initial cohort management strategy will follow a standard single-dose escalation design and enrol patients with ovarian cancer and advanced solid tumours. This approach will allow INNOVOTEX to identify exploratory biomarkers that can serve as enrolment criteria for the expansion cohorts that will ensue following an initial Phase I clinical study. This expansion and the associated data, if favourable, will help set the scene for strategic partnerships. INNOVOTEX currently anticipates an approval pathway for OxaliTEX (5) that will evolve from second-line therapy to combination use, with the end goal of creating a new standard of care for front-line therapy. Such an outcome, representing the culmination of success through multiple preclinical, clinical, and regulatory steps, is viewed as being highly desired by clinicians and patients. It would allow INNOVOTEX to address an urgent and unmet clinical need directly while setting the stage for the expansion of the TEX Core strategy into other cancer disease areas.

Currently, INNOVOTEX is in the midst of a seed financing round. Proceeds of this planned raise will be used to finance the manufacturing of OxaliTEX (5), fund pre-clinical new drug studies, including advanced toxicological work. The funds will also be used to maintain and expand INNOVOTEX’s IP portfolio as in-licensed from UT Austin or developed in house. The dominant focus of these efforts will be to bring OxaliTEX (5) into the clinic as an experimental drug targeting platinum-resistant ovarian cancer. This is an orphan disease that constitutes an unmet medical need and one that benefits from a perceived favourable regulatory pathway.

The INNOVOTEX team is no stranger to the devastating role of cancer in our personal lives. As such, we are humbled to be at the forefront of therapeutic innovation. The ability to leverage clinically-validated data and redesign texaphyrin chemistry has derisked our development program and clinical strategy. We are excited to embark on the next chapter of company growth with a phenomenal team, passionate about creating innovative therapies and improving quality of life for patients with advanced cancers.

## Author contributions

All authors were involved in the preparation of the manuscript, as indicated in each section heading. The final manuscript was approved by all authors.

## Conflicts of interest

APD is a founder and shareholder of Carbometrics. JFA, KK and JLS are co-founders of INNOVOTEX, Inc. and have an equity stake in the company. The remaining authors have no conflicts of interest to report.

## Data Availability

No primary research results, software or code have been included and no new data were generated or analysed as part of this editorial.
